# 腰大肌旁多形性脂肪肉瘤复发伴纵隔转移1例及文献回顾

**DOI:** 10.3779/j.issn.1009-3419.2017.05.10

**Published:** 2017-05-20

**Authors:** 京豪 刘, 作庆 宋, 仁旺 刘, 明辉 刘, 典 任, 清华 周, 军 陈

**Affiliations:** 300052 天津，天津医科大学总医院肺部肿瘤外科，天津市肺癌研究所，天津市肺癌转移与肿瘤微环境实验室 Department of Lung Cancer Surgery, Tianjin Medical University General Hospital, Tianjin Key Laboratory of Lung Cancer Metastasis and Tumor Microenvironment, Tianjin Lung Cancer Institute, Tianjin Medical University General Hospital, Tianjin 300052, China

**Keywords:** 纵隔转移性肿瘤, 多形性脂肪肉瘤, 多学科综合治疗, Metastatic mediastinal tumor, Pleomorphic liposarcoma, Multidisciplinary therapy

## Abstract

纵隔内转移性多形性脂肪肉瘤为纵隔内肿瘤的罕见病种，目前总体治疗效果欠佳。本文介绍腰大肌旁多形性脂肪肉瘤复发伴纵隔转移1例，探讨多形性脂肪肉瘤的临床特点和治疗策略。回顾性分析我院收治的1例转移性多形性脂肪肉瘤患者，基于影像学诊断与手术病理诊断，对患者进行针对性的多学科综合治疗。一名41岁女性患者，诊断腰大肌旁多形性脂肪肉瘤伴纵隔转移。经手术、靶向及化疗等多学科治疗，随访至今已65个月。对于多形性脂肪肉瘤治疗策略，应以外科完整切除为首选，术后辅助相关内科治疗，多学科综合治疗以延缓患者病情进展及复发。

## Abstract (en)

Metastatic pleomorphic liposarcoma in the mediastinum is rare, and the current treatments are most of the times ineffective. We hereby report a case with relapsed pleomorphic liposarcoma adjacent to the psoas major muscle and with mediastinal metastasis, to discuss the clinical features and treatment strategies of pleomorphic liposarcoma. The patient's clinical history, imaging findings, pathological diagnosis, and multidisciplinary treatments were retrospectively analyzed. A 41-year old female patient was diagnosed with pleomorphic liposarcoma adjacent to the psoas major muscle and mediastinal metastasis. After multidisciplinary treatments, including surgery, chemotherapy, and targeted therapy, the patient has survived 65 months until now. Surgical resection is considered as the first choice of the treatment for pleomorphic liposarcoma. Multidisciplinary treatments, including chemotherapy and other medical treatments, are effective to slow the disease progression and to reduce the disease recurrence.

脂肪肉瘤为成人较少见病例，约占人类恶性肿瘤的1%^[1]^，约占成人软组织肉瘤的20%，其中多形性脂肪肉瘤更为罕见，约占所有脂肪肉瘤的5%^[2, 3]^。查阅国内外相关文献，笔者发现脂肪肉瘤原位复发伴纵隔转移的相关个案报道甚少，妊娠期复发转移病例尚无相关报道。我院收治一例妊娠期腰大肌旁多形性脂肪肉瘤肉瘤复发伴纵隔转移患者，现将其临床资料进行回顾性分析，并复习相关文献讨论如下。

## 1 病例介绍

患者，女性，41岁，2011年5月因发现右腰部一进行性增大肿物一月就诊。查体示右腰部可及一约6 cm×8 cm肿物，质硬，边界较清，轻压痛。完善相关检查，磁共振成像（magnetic resonance imaging, MRI）提示右腹膜后肿物，可见不均匀强化，病灶边缘可见不规则明显强化，其内可见大小不等点状无强化区及线样分隔，与腹膜边界不清，考虑恶性软组织瘤。后行手术治疗，术中探查提示腹膜后、腰大肌旁可见一10.5 cm×8.5 cm×4.5 cm肿物，质硬，成膨胀性生长。包膜较完整，与部分腰大肌粘连。经手术完整切除肿物，术后病理回报：多形性脂肪肉瘤。术后患者顺利出院。

2013年7月患者无意间发现右腰部原手术部位一肿物进行性生长，遂于门诊行腰大肌MRI平扫，提示右腰大肌旁可见一等T1、稍长T2信号为主混杂信号类圆形囊实性肿物，其内可见多发类圆形小囊性长T1、长T2信号。边界较清楚，大小约90.7 cm×64.5 cm×78.2 mm，与腰大肌关系密切。患者存在胸痛症状，行胸部CT检查提示右上纵隔占位，半月后复查复计算机断层扫描（computed tomography, CT）提示占位进行性增大，与上纵隔边界不清（图 1）。收治入院后，完善全身检查，提示除腰部及纵隔占位外未见其他明确转移。妇科B超提示患者早孕（相当于孕8+周），行人工流产术。考虑同期切除纵隔及腰部肿瘤创伤较大，顾先行纵隔肿瘤切除，待患者恢复后行腰部肿瘤切除。全麻下常规右后外侧开胸探查提示：右上纵隔来源一巨大肿球形肿物，直径约10 cm，包膜较完整（图 1）。与右上肺关系紧密，富血供粘连带相连。右上肺受压明显。完整切除后剖开肿瘤切面示灰黄、灰白相间，质韧偏软，切面细腻。送检病理汇报多形性脂肪肉瘤。患者纵隔肿瘤切除前，存在畏寒情况，反复出现感冒症状，白细胞可升至10×10^9^个/L，使用头孢西丁症状可明显缓解。纵隔肿瘤切除后，此情况未再发生。术后患者恢复顺利（图 2），1月后骨科就诊，行手术切除腰部肿瘤。术中发现腹膜后、腰大肌旁一球形肿物，直径约13 cm，与2011年手术切除肿瘤位置大致相同，表面有假膜，可见迂曲静脉分布。完整切除送检，病理再次回报：多形性脂肪肉瘤，包膜完整。术后患者恢复较为顺利。患者恢复阶段，对患者切除肿瘤行基因检测，提示患者存在表皮生长因子受体（epidermal growth factor receptor, EGFR）突变19外显子突变。术后两月患者口服吉非替尼，随诊观察6个月，患者病情缓慢进展，患者自行停药。1年后患者再次就诊，右上纵隔病灶较前略有增大，故行6周期化疗，方案：表阿霉素（d1）+异环磷酰胺（d1-d5）+长春地辛（d1）。至2016年10月，患者病灶仍略有进展，患者仍在随访中。

**1:**
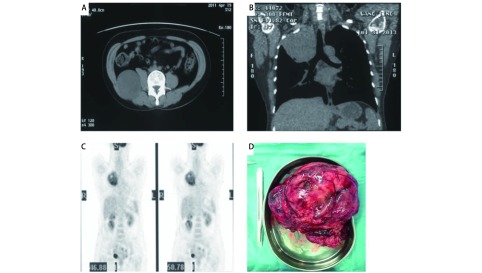
患者2013年纵隔肿物切除前影像资料及纵隔肿物大体标本。A:患者2013年腰大肌旁肿瘤复发；B：2013年右上纵隔转移，胸部冠状位CT；C：两处病变PET-CT提示高代谢；D：纵隔肿瘤完整切除大体标本。 The image data before the resection of the mediastinal tumor in 2013 and the macroscopic view of the mediastinal tumor. A: Relapsed pleomorphic liposarcoma adjacent to the psoas major muscle in 2013; B: The chest coronary CT scan demonstrates right upper mediastinal metastasis in 2013; C: PET-CT scan indicates hypermetabolism in the psoas major muscle and mediastinum. D: Macroscopic view of the completely resected tumor in the mediastinum. PET-CT: positron emission tomographycomputed tomography.

**2:**
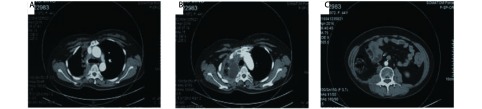
患者2013年术后影像检查。A：胸部CT提示：经手术后患者纵隔内肿物完整切除；B：胸部CT提示：经手术后患者纵隔内肿物完整切除；C：腰大肌旁肿物切除后肿物消失。 CT scan after the surgery in 2013. A: Chest CT indicates mediastinal tumor was completely resected. B: Chest CT indicates mediastinal tumor was completely resected. C: CT scan indicates tumor adjacent to the psoas major muscle was completely resected.

**3:**
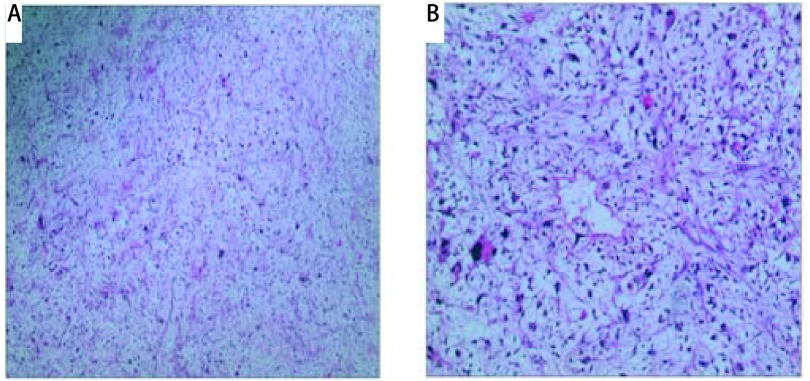
标本电镜下照片，均考虑多形性脂肪肉瘤。A：电镜图片（×100）；B：电镜图片（×400）。 Pathological examination revealing the pleomorphic liposarcoma cells. A: Microscopy magnification, ×100; B: Microscopy magnification, ×400.

## 2 讨论

多形性脂肪肉瘤为脂肪细胞来源的恶性肿瘤。依据WHO2013软组织肿瘤分类将恶性脂肪细胞分为以下五类：去分化脂肪肉瘤（dedifferentiated liposarcoma）、粘液样脂肪肉瘤（myxoid liposarcoma）、多形性脂肪肉瘤（pleomorphic liposarcoma）、混合型脂肪肉瘤（mixed-type liposarcoma）、脂肪肉瘤，未分类脂肪肉瘤（liposarcoma, not otherwise specified）^[3]^。其中多形性脂肪肉瘤最为罕见，约占所有脂肪肉瘤的5%^[2]^，相关的大宗病例报道较少^[4-6]^。作为脂肪肉瘤的一种，多形性脂肪肉瘤，大多性情况下为无痛性进行生长的肿物，常见于老年患者，好发于下肢，盆腔、腹壁、胸壁、肠系膜、腹膜、精索和腹膜后较为少见，纵隔内生长罕见^[7]^。多形性脂肪肉瘤具有最高的侵袭性, 总体转移几率约为30%-50%，最易发生肺内转移，其次是肝和骨^[2, 6-8]^。确诊为局限性病灶的患者，5年生存率约为60%。疾病复发转移、手术不能切除、及切缘阳性都是预后不良的独立预测因子^[9-11]^。

在本病例中，该患者病情较一般转移更具个体性，首先患者为中年女性，确诊多形性脂肪肉瘤2年后出现复发转移，转移部位为纵隔，在笔者查阅的国内外文献中尚无纵隔转移的相关报道。其次，患者出现明显肿瘤复发、转移时间与其妊娠时间基本一致。推测妊娠可能与多形性脂肪肉瘤的转移复发存在一定相关性，这可能与富血供状态相关。

关于多形性脂肪肉瘤的诊断，目前认为X线对肿瘤的诊断作用甚小，仅在肺转移时对诊断有一定帮助。对于本病例X线仅能提示纵隔占位。分化良好的脂肪肉瘤, 肉眼上可见脂肪来源肿瘤，影像学表现为以脂肪为主的不均质肿块，CT和MRI较易显像^[12]^。因此CT和MRI对于术前评估及鉴别诊断起到更重要的作用。CT能更好的提示肿瘤骨破坏及肿瘤钙化的情况，而MRI是在描述脂肪肿瘤的性质和分类上更具优势^[13]^。对于本病例，正电子发射型计算机断层显像（positron emission tomography-computed tomography, PET-CT）很好地提示了肿瘤的复发以及转移，但目前PET-CT对于脂肪肉瘤诊断作用仍在探索中，仅个别个案报道了其对脂肪肉瘤具有诊断作用^[14-16]^。因此PET-CT对于多形性脂肪肉瘤的诊断作用仍有待研究。

完整的手术切除毫无疑问是多形性脂肪肉瘤的首选治疗方法。能否完整切除直接影响到患者的预后。完整切除后肿瘤复发的概率为1%-10%，但肿瘤或肿瘤包膜的残留，可使复发几率提升至40%-100%^[17]^。目前考虑较安全的切除范围为2 cm，但一般多形性脂肪肉瘤生长于躯体深部，这也就增大了扩大切除的难度。纵隔肿瘤的切除难度大，复发率远大于其他部位^[18, 19]^。

多形性脂肪肉瘤患者发生复发转移后，其预后要显著差于局部肿瘤患者。Markus等^[20]^回顾性研究了155例多形性脂肪肉瘤患者。研究发现，多形性脂肪肉瘤患者发生远处转移中位时间，为确诊后8.5个月（2.5个月-47.2个月），转移后患者中位总生存期为9.1个月，1年生存率45%，无5年生存患者。相较于肿瘤大小、病理分级和肿瘤是否原发这些因素而言，手术切缘阳性和放化疗似乎与患者生存期有着更密切的关系。

放疗是仅次于手术的治疗手段，主要用于无法手术或术后存在残留的情况，但其疗效目前仍存在争议。公认粘液样脂肪肉瘤具有最好的疗效^[21]^。有学者提出应将脂肪肉瘤视为全身性疾病，可使用化疗药物治疗。但由于多形性脂肪肉瘤发病率低，缺乏大宗临床数据，目前尚无标准化疗方案，即使化疗可使46%的肿瘤进展患者存在一定的临床获益，但生存期仍然很低^[22]^。本例患者在进行基因检测后，存在EGFR基因突变，使用靶向药物吉非替尼治疗，后又进行系统性化疗，疾病进展得到了较有效的控制。目前该患者规律复查，总生存期已达65个月，远高于相关文献报道^[23]^。笔者认为完整手术切除和术后系统性化疗，明显提高了该患者的生存时间。

总而言之，本案例所报道的妊娠期腰大肌旁多形性脂肪肉瘤复发伴纵隔转移为罕见病例。疾病的初诊应充分结合影像学检查结果，对已明确的肿物进行诊断同时，仍应对全身进行评估，从而避免漏诊。尽早行手术治疗，争取扩大完整切除。术后适量配合放化疗，可使患者获得一定的受益。对于术后患者做好随诊工作，尽早对肿瘤的复发及转移进行处理。结合本病例，两点值得我们思考。第一，随着基因检测的普及，越来越多的靶点基因突变结果来自间质性肉瘤病例，但这部分患者靶向药物应用的效果仍缺乏大宗临床数据证实。第二，虽目前考虑怀孕及哺乳会影响脂肪肉瘤的病情，但仍缺乏相关研究证实这一观点。是否能建议罹患该类型肿瘤的女性患者适当避孕，从而更好地治疗及康复，仍有待证实。

## References

[1] Dei Tos AP (2000). Liposarcoma: new entities and evolving concepts. Ann Diagn Pathol.

[2] Wang L, Ren W, Zhou X (2013). Pleomorphic liposarcoma: a clinicopathological, immunohistochemical and molecular cytogenetic study of 32 additional cases. Pathol Int.

[3] Dodd LG, Sara Jiang X, Rao K (2015). Pleomorphic liposarcoma: a cytologic study of five cases. Diagn Cytopathol.

[4] Gebhard S, Coindre JM, Michels JJ (2002). Pleomorphic liposarcoma: clinicopathologic, immunohistochemical, and follow-up analysis of 63 cases: a study from the French Federation of Cancer Centers Sarcoma Group. Am J Surg Pathol.

[5] Hornick JL, Bosenberg MW, Mentzel T (2004). Pleomorphic liposarcoma: clinicopathologic analysis of 57 cases. Am J Surg Pathol.

[6] Dalal KM, Kattan MW, Antonescu CR (2006). Subtype specific prognostic nomogram for patients with primary liposarcoma of the retroperitoneum, extremity, or trunk. Ann Surg.

[7] Oliveira AM, Nascimento AG (2001). Pleomorphic liposarcoma. Semin Diagn Pathol.

[8] Downes KA, Goldblum JR, Montgomery EA (2001). Pleomorphic liposarcoma: a clinicopathologic analysis of 19 cases. Mod Pathol.

[9] de Moraes FB, Cardoso AL, Tristão NA (2015). Primary liposarcoma of the lumbar spine: case report. Rev Bras Ortop.

[10] Hamlat A, Saikali S, Gueye EM (2005). Primary liposarcoma of the thoracic spine: case report. Eur Spine J.

[11] Saeed M, Plett S, Kim GE (2010). Radiological-pathological correlation of pleomorphic liposarcoma of the anterior mediastinum in a 17-year-old girl. Pediatr Radiol.

[12] Nevile A, Herts BR (2004). CT characteristics of p rimary retroperitonealneop lasms. Crit Rev Comput Tomogr.

[13] Kransdorf MJ, Bancroft LW, Peterson JJ (2002). Imaging of fatty tumors: Distinction of lipoma and well differentiated liposarcoma. Radiology.

[14] Yang J, Codreanu I, Servaes S (2012). Earlier detection of bone metastases from pleomorphic liposarcoma in a pediatric patient by FDG PET/CT than planar 99mTc MDP bone scan. Clin Nucl Med.

[15] Kudo H, Inaoka T, Tokuyama W (2012). Round cell liposarcoma arising in the left foot: MRI and PET findings. Jpn J Radiol.

[16] Lee SA, Chung HW, Cho KJ (2013). Encapsulated fat necrosis mimicking subcutaneous liposarcoma: radiologic findings on MR, PET-CT, and US imaging. Skeletal Radiol.

[17] Sezer A, Tuncbilek N, Usta U (2009). Pleomorphic liposarcoma of the pectoralis major muscle in an elderly man: report of a case and review of literature. J Cancer Res Ther.

[18] Macchiarini P, Ostertag H (2004). Uncommon primary mediastinal tumours. Lancet Oncol.

[19] Boland JM, Colby TV, Folpe AL (2012). Liposarcomas of the mediastinum and thorax: a clinicopathologic and molecular cytogenetic study of 24 cases, emphasizing unusual and diverse histologic features. Am J Surg Pathol.

[20] Ghadimi MP, Liu P, Peng T (2011). Pleomorphic liposarcoma: clinical observations and molecular variables. Cancer.

[21] Yu L, Jung S, Hojnowski L (2005). Best cases from the AFIP: Dedifferentiated liposarcoma of soft tissue with high-grade osteosarcomatous dedifferentiation. Radiographics.

[22] Italiano A, Toulmonde M, Cioffi A (2012). Advanced well-differentiated/ dedifferentiated liposarcomas: role of chemotherapy and survival. Ann Oncol.

[23] Chen M, Yang J, Zhu L (2014). Primary intrathoracic liposarcoma: a clinicopathologic study and prognostic analysis of 23 cases. J Cardiothorac Surg.

